# Monitoring of indoor bioaerosol for the detection of SARS-CoV-2 in different hospital settings

**DOI:** 10.3389/fpubh.2023.1169073

**Published:** 2023-04-20

**Authors:** Emma Tedeschini, Stefania Pasqualini, Carla Emiliani, Ettore Marini, Alessandro Valecchi, Chiara Laoreti, Stefano Ministrini, Barbara Camilloni, Roberto Castronari, Lucio Patoia, Francesco Merante, Stefano Baglioni, Edoardo De Robertis, Matteo Pirro, Antonella Mencacci, Leonella Pasqualini

**Affiliations:** ^1^Dipartimento di Scienze Agrarie, Alimentari e Ambientali, Università degli Studi di Perugia, Perugia, Italy; ^2^Dipartimento di Chimica, Biologia e Biotecnologie, Università degli Studi di Perugia, Perugia, Italy; ^3^Dipartimento di Medicina e Chirurgia, Università degli Studi di Perugia, Perugia, Italy; ^4^Center for Molecular Cardiology, University of Zurich, Zurich, Switzerland; ^5^Ospedale S.G. Battista – Azienda Unità Sanitaria Umbria 2, Foligno, Italy; ^6^S.C. Pneumologia, Ospedale Santa Maria della Misericordia, Azienda Ospedaliera di Perugia, Perugia, Italy

**Keywords:** SARS-CoV-2, bioaerosol, aerobiology, environmental monitoring, environmental prevention

## Abstract

**Background:**

Spore Trap is an environmental detection technology, already used in the field of allergology to monitor the presence and composition of potentially inspirable airborne micronic bioparticulate. This device is potentially suitable for environmental monitoring of Severe Acute Respiratory Syndrome Coronavirus 2 (SARS-CoV-2) in hospital, as well as in other high-risk closed environments. The aim of the present study is to investigate the accuracy of the Spore Trap system in detecting SARS-CoV-2 in indoor bioaerosol of hospital rooms.

**Methods:**

The Spore Trap was placed in hospital rooms hosting patients with documented SARS-CoV-2 infection (*n* = 36) or, as a negative control, in rooms where patients with documented negativity to a Real-Time Polymerase Chain Reaction molecular test for SARS-CoV-2 were admitted (*n* = 10). The monitoring of the bioaerosol was carried on for 24 h. Collected samples were analyzed by real-time polymerase chain reaction.

**Results:**

The estimated sensitivity of the Spore Trap device for detecting SARS-CoV-2 in an indoor environment is 69.4% (95% C.I. 54.3-84.4%), with a specificity of 100%.

**Conclusion:**

The Spore Trap technology is effective in detecting airborne SARS-CoV-2 virus with excellent specificity and high sensitivity, when compared to previous reports. The SARS-CoV-2 pandemic scenario has suggested that indoor air quality control will be a priority in future public health management and will certainly need to include an environmental bio-investigation protocol.

## 1. Introduction

Severe Acute Respiratory Syndrome Coronavirus 2 (SARS-CoV-2) is transmitted through contaminated aerosols, released by infected individuals (1, 2). Viral particles are encapsulated in droplets of mucus, saliva, and water, which can travel through the environment in air suspension. The fate of droplets in the environment depends on their size. Large droplets fall to the ground soon after their emission, without evaporating. Indeed, droplets larger than 100 μm typically fall to the ground within 2 m from the source and can be sprayed only to nearby individuals; for this reason, physical distancing is a pivotal measure to prevent contagion by airborne pathogens (3).

On the other hand, droplets smaller than 100 μm can also travel longer than 2 m from the source. They can stay in air suspension for hours and are highly concentrated in the nearby of infected patients; furthermore, they can accumulate in poorly ventilated closed spaces, constituting a high-risk setting for viral transmission (4, 5). Therefore, the scientific community has established a particularly restrictive prevention protocol for indoor environments, where the highest concentration of small droplets can be found.

Besides established preventive measures, the possibility to detect the presence of SARS-CoV-2 in indoor environments, could help identifying subjects exposed to the pathogen, and adopting targeted preventive measures.

The Spore Trap environmental detection technology, already used in the field of allergology, monitors the presence and composition of potentially inspirable airborne micronic bioparticulate (pollen and fungal spores). For this purpose the machine aspirates a volume of air that corresponds to usual human pulmonary ventilation (6–8).

This technology, consisting of a microscope slide moving over a slit, was first designed in 1,952, and it successfully measured the total concentrations of spores and pollen in the atmosphere for each hour of the day. Currently used volumetric samplers, including the Burkard spore trap and the Lanzoni sampler, are based on this same design. Samplers generally operate continuously over a time lapse 1–7 days, and have a broad range of applications for indoor air sampling (9–11). The samples are usually analyzed under an optical microscope to identify visible bioparticulate. Recently, the application of molecular biology protocols significantly increased the sensibility and resolution of this method, allowing to analyze the aerosolized submicronic microbiota too (12).

The aim of the present study is to investigate the accuracy of the Spore Trap system in detecting SARS-CoV-2 in indoor bioaerosol of hospital rooms.

## 2. Materials and methods

### 2.1. Study design

All patients were tested for SARS-CoV-2 infection with PCR on nasopharyngeal swab at the hospital admission. Patients who resulted negative were checked again after 7 days, as per regulation in the Umbria region. The environmental monitoring was started within 24 h after the hospital admission. The Spore Trap device was placed in hospital rooms hosting patients with documented SARS-CoV-2 infection (*n* = 36) or, as a negative control, in rooms where patients with documented negativity to a PCR molecular test for SARS-CoV-2 were admitted (*n* = 10).

**Figure 1:**
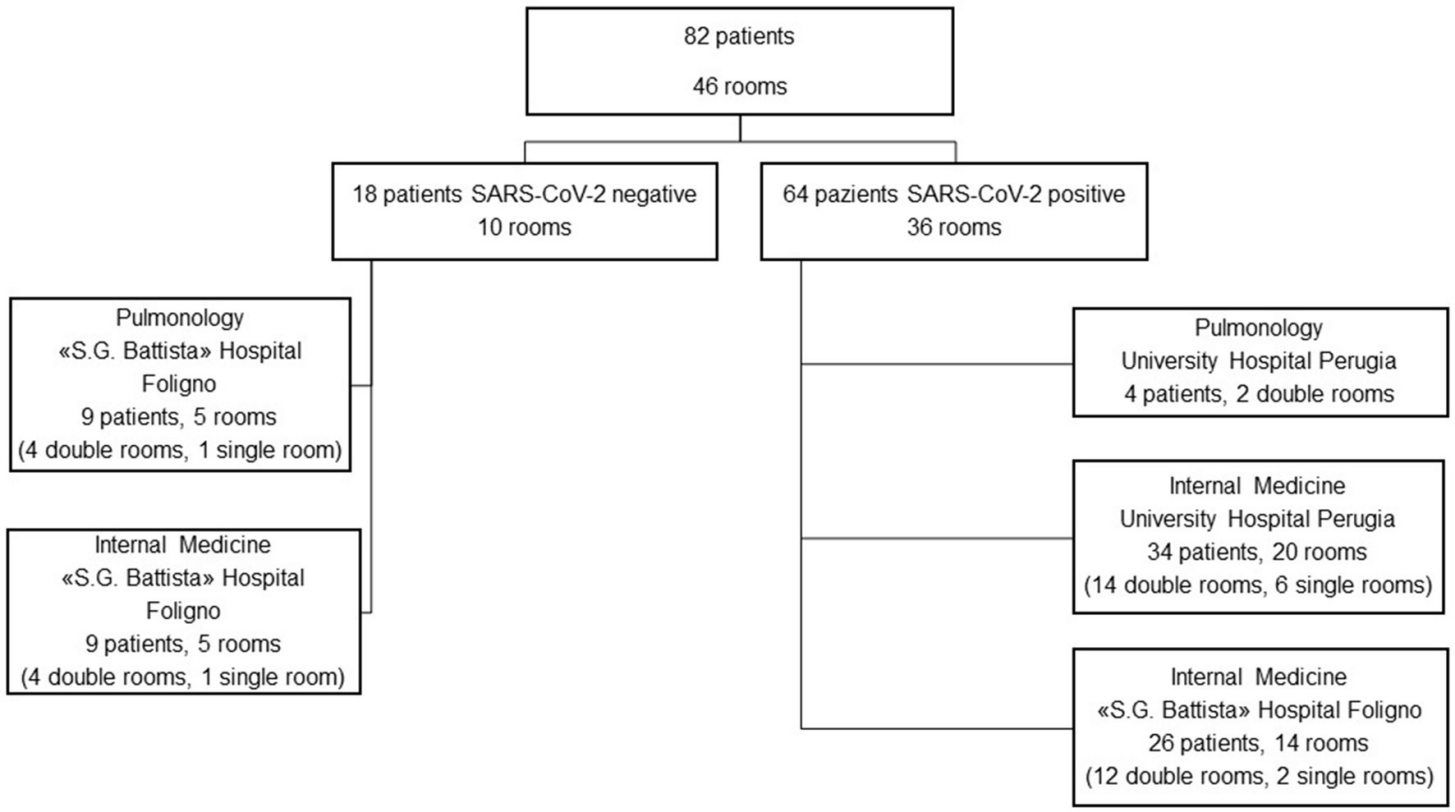
Flow-chart of the study design.

Measurements were conducted between March and June 2021 in two hospitals of the Umbria region (Italy): University Hospital “Santa Maria della Misericordia” of Perugia and “S.G. Battista” Hospital in Foligno (PG, Italy). SARS-CoV-2-dedicated spaces were in both hospitals, whereas the control spaces were located at the “S.G. Battista” Hospital only (Figure 1). Patients were single-roomed or double-roomed, and received standard oxygen therapy, non-invasive ventilation, or no respiratory support according to clinical necessities. Rooms had a mechanical ventilation system under atmospheric pressure. Subjects included in the study were aged at least 18 years old, hospitalized for any cause. No exclusion criterion was adopted for the SARS-CoV-2 positive patients, whereas patients in the control group were excluded if they turned out positive within 5 days from the environmental measurement (no case).

Informed consent to participate in the study was acquired in compliance with the provisions of the EU Guidelines for Good Clinical Practice (DM 15th July 1997). The procedures required to acquire the data were in accordance with the ethical principles contained in the 1964 Declaration of Helsinki. The identification data of the subjects involved in this research (name, surname, date and place of birth, place of residence) have been made anonymous for storage and processing, in accordance with EU Regulation 2016/679. The study protocol was approved by the Ethical Commission of the University of Perugia (nr. 61812/2021).

### 2.2. Collected parameters

For each measurement, the following parameters were recorded: number of patients in the environment, PCR results of the nasopharyngeal swab for SARS-CoV-2 RNA, and use of respiratory support. General demographic and clinical characteristics of patients were also collected.

### 2.3. Sampling strategy

In each room the monitoring of the bioaerosol was carried out for 24 h using a Spore Trap air sampler (VPPS−1000 Lanzoni) for indoor biomonitoring.

The machine contains an electric vacuum pump that aspires a pre-determined volume of air (10 L/min) from the external environment. The flow of air is sucked through a slit (2 × 14 mm) and hits the sampling surface (2 × 48 mm slide), placed on a moving slide. The slide moves at 2 mm/h speed, so that 2 mm of the surface is exposed to the air flow for 1 h. The sampling surface was treated with an experimental solution [10% Poly-D-lysine (PDL) and poly-L-lysine (PLL) in Guanidine Hydrochloride Buffer Solution, 6 mol/L, pH 8,7] to improve adhesion and adsorption.

Considering the source of the bioaersol (patients in a constantly lying position) and the quality of the bioparticulate (dispersion by droplets), the device was positioned about 130–140 cm from the ground, in the corner of single rooms or equidistant between two beds in double rooms.

Collected samples were scraped off the slides and treated with lysis buffer. The RNA was extracted in a QIAsymphony^®^ SP Workstation using the QIAsymphony^®^ DSP Virus/Pathogen Kit (QIAGEN, Milan, Italy), in accordance with the manufacturer's instructions. Real time PCR (RT-PCR) was carried using the Thermo Fisher^®^ TaqPath™ COVID19 CE-IVD RT-PCR kit and the QuantStudio™ 5 Real-Time PCR System. The TaqPath assay targets three sequences in the virus ORF1ab, N and S genes. The internal control for nucleic acid extraction was an MS2 phage. Reverse transcription was carried out at 53°C for 10 min, pre-denaturation at 95°C for 2 min followed by 40 cycles of denaturation at 95°C for 3 s and annealing at 60°C for 30 s. To quantify the RNA, dilutions of PCR-kit positive control (10-1-10-4) were used. Results were interpreted using the COVID19 Interpretive Software version v.2.5 on QuantStudio™ Design and Analysis Desktop Software v.1.5.1.

### 2.4. Statistical analysis

Data are expressed as mean (SD) for continuous variables and as number (%) for categorical variables. Significance of analyses is set at <0.05 for type I error. Comparisons are performed by Student's *t*-test and χ^2^ test for continuous and categorical variables, respectively. Sensitivity (Se) is calculated as $Se=\frac{True\;Positive}{\left(True\;Positive+False\;Negative\right)}$. Specificity (Sp) is calculated as $Sp=\frac{True\;Negative}{\left(True\;Negative+False\;Positive\right)}$. For each indicator, a 95% confidence interval (95% C.I.) was calculated as follows: $p\pm 1.96\times \sqrt{\frac{p\left(1-p\right)}{n}}$, where p represents the indicator and n is the sample size. Data were analyzed using GraphPad Prism 9.5.1 (GraphPad Software Inc. Boston, MA, USA).

## 3. Results

**Figure 2:**
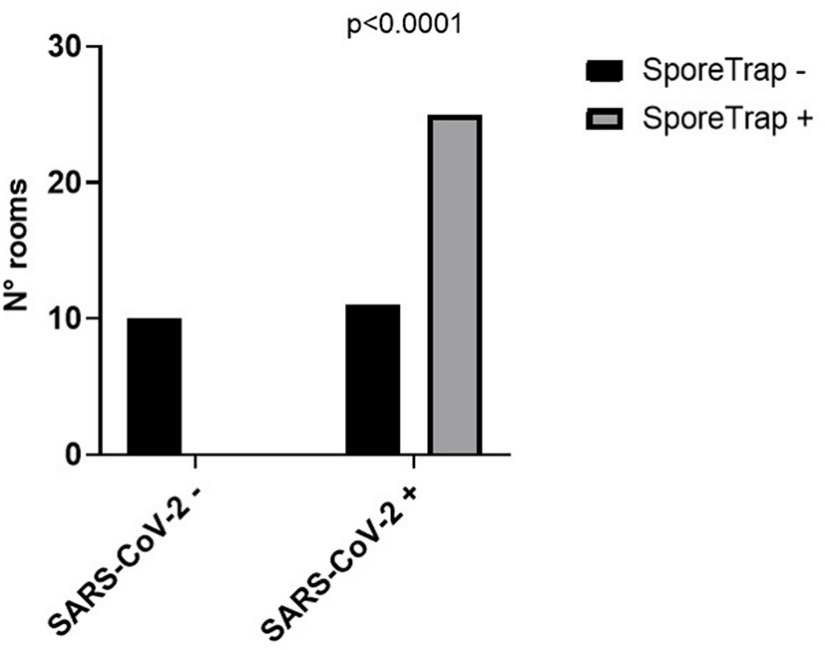
Performance of the SporeTrap device in detecting the presence of Severe Acute Respiratory Syndrome Coronavirus 2 (SARS-CoV-2) in contaminated (SARS-CoV-2 +) and non-contaminated (SARS-CoV-2 -) hospital rooms.

The characteristics of the involved patients are reported in Table 1. SARS-CoV-2 RNA was not detected in the ten control rooms, whereas it was detected in 25 out of 36 SARS-CoV-2 rooms (Figure 2). So, the estimated sensitivity of the Spore Trap device for detecting SARS-CoV-2 in an indoor environment is 69.4% (95% C.I. 54.3–84.4%), with a specificity of 100%.

**Table 1:** Characteristics of subjects enrolled in the study according to the SARS-CoV2 status.

**Parameter**	**SARS-CoV-2 negative**	**SARS-CoV-2 positive**	***p***
Age (yrs)	79.6 (11.9)	70.0 (14.3)	0.006
Sex (females)	8 (42.1)	39 (60.9)	0.146
Onset of symptoms to swab time (days)	16.9 (20.2)	17.1 (14.4)	0.971
nTC gene N	-	28 (25–31)	-

nTC, number of thermic cycles; SARS-CoV-2, Severe Acute Respiratory Syndrome Coronavirus 2.

**Figure 3:**
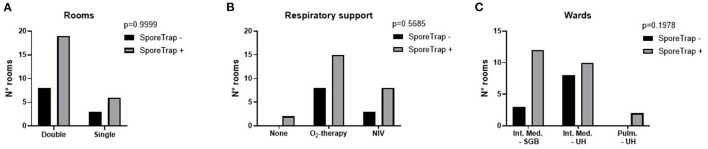
Possible factors influencing the detection of Severe Acute Respiratory Syndrome Coronavirus 2 (SARS-CoV-2) by the SporeTrap technology. **(A)** Double rooms vs. single rooms. **(B)** Presence and modality of respiratory support. **(C)** Site of detection. NIV, non-invasive ventilation; SGB, “San Giovanni Battista” Hospital; UH, University Hospital.

**Figure 4:**
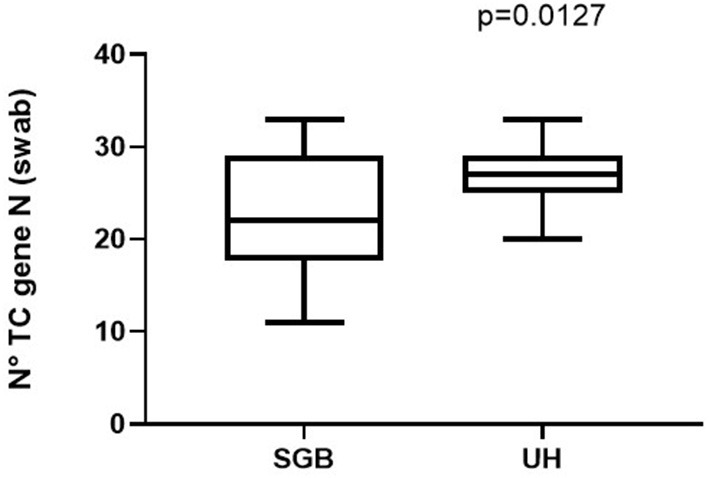
Different viral load reported in patients admitted to the University Hospital (UH) or to the “San Giovanni Battista” Hospital (SGB).

Then we explored the potential factors influencing the sensitivity of the measure: no significant difference was observed between single and double rooms (Figure 3) and among different methods of respiratory support (Figure 3B). Although non-significant, an unexpected difference was observed between the two hospitals (Figure 3C). Raw data are reported in the Supplementary Table 1. Comparing the number of thermic cycles (nTC) of the PCR test on nasopharyngeal swab, patients had an average lower nTC in the “S.G. Battista” hospital than in the University hospital (Figure 4), consistent with the different sensitivity of the method observed in the two sites.

## 4. Discussion

The environmental monitoring performed with the Spore Trap method has shown a high specificity in excluding the presence of SARS-CoV-2 in non-contaminated environments, with a good sensitivity in detecting the virus in contaminated environments.

Previous attempts to detect the presence of the virus in healthcare institutions yielded conflicting results (13), although it has been previously demonstrated that it is possible to detect microbial contamination in high-risk environments, by using an appropriate sampling protocol and a correct detection method (14). In this regard, the main issue is represented by the high variability of virus concentration in the air, depending on several factors: viral load emitted by sources, air clearance by ventilation, air dynamics in the environment, etc. As a result, to the present day, no method can reliably measure the concentration of viral particles in the air. To overcome this technical issue, we adopted a pragmatic study design, based on the estimation of the environmental biological risk, rather than on the measurement of viral particles in the air. To this end, the negative control was constituted by rooms inhabited by SARS-CoV-2 negative subjects, who remained negative for the subsequent 5 days after the air sampling. These environments were assumed to have a very-low risk of biologically relevant contamination from SARS-CoV-2 at the time of sampling. On the other hand, the positive control was constituted by rooms inhabited by SARS-CoV-2 patients, who resulted positive during the previous 24 h with a low/intermediate nTC at the nasal swab, as reported in Table 1. Since the ventilation system in the rooms operated under atmospheric pressure, these environments were judged to have a very-high risk of biologically relevant contamination from SARS-CoV-2 at the time of sampling.

The detection of airborne viruses in the environment may use different technologies, including gravitational deposition, solid impact, liquid impinging, filtration, and aspiration with varying efficacy in detecting SARS-CoV-2 (15). Previous studies indicated that solid impactors are more effective than liquid impingers or filters. Furthermore, additional parameters may influence the performance of a bioaerosol sampler, including the distance between the device and the source, its height from the floor, the flow rate and the volume of air sampled (13).

In this regard, the Spore Trap technology has multiple strength points: firstly, it employs a hybrid technology combining solid impact and aspiration, which allows to effectively process a large volume of air. Furthermore, by performing a 24-h monitoring, it allows to process a larger volume of air than short-time samplings (4, 16). In particular, the device processes a volume of air that corresponds to usual human pulmonary ventilation, providing a reliable risk estimate for detected particles to be inhaled by a person dwelling in that environment. The Spore Trap is designed for capturing large particles, like pollens and fungal spores; however, it could also capture large fluid droplets, containing viral particles, before evaporation. Furthermore, SARS-CoV-2 has been observed to associate to micronic bio-aerosol (17), then the Spore Trap technology could detect the virus associated to pollens and fungal spores, which constitute a component of environmental air where the virus is particularly concentrated.

At the best of our knowledge, three previous studies reported similar sensitivity in detect-ing SARS-CoV-2 in air samples from contaminated environments (66.7, 54.3, and 38.7%, respectively). In the study of Chia et al. (18) a NIOSH BC 251 bioaerosol sampler was employed, using a hybrid technology similar to the Spore Trap device, with the additional feature of separating particles by diameter (>4 μm, 1–4 μm, and <1 μm). The aspiration rate was set at 3.5 L/min and run for 4 h, collecting a total of 5040 L of air from each patient's room. However, the study included only 3 patients, so it could be considered as a proof-of-concept only. In the study of Liu et al. (19) three different sampling methods were employed, namely filtration with aspiration, cascade impactor and gravitational deposition. Although measurements were performed in two hospitals dedicated to SARS-CoV-2 positive patients, sampled environments had different risk of contamination (e.g., intensive care units with SARS-CoV-2 patients and medical staff rooms). Furthermore, different environments were sampled with different methods, so that it is not possible to estimate the detection performance of a specific method. Finally, Zhou et al. (20) employed a hybrid technology with aspiration and liquid impact. Like Liu et al. (19) they also sampled environments with different risk of contamination. All these studies were conducted in the very early phases of the pandemic, and they aimed at confirming the airborne transmission of SARS-CoV-2. None of them included a negative control group. In this regard, it is important to underscore that, in our study, none of the patients in the control group turned positive to SARS-CoV-2 in the 5 days following the measurement. This confirms that no clinically relevant contamination of the environment occurred at the time of the measurement.

Conversely, our study was controlled to test a potential practical use of environmental monitoring to prevent SARS-CoV-2 diffusion. Our data prove that the detection of airborne viruses by the Spore Trap device is specific and can be successfully applied to a real-world setting. Indeed, although the sensitivity of our protocol is still sub-optimal, it is yet the highest described so far. However, no comparative study has been performed yet.

The possibility to extend the use of environmental monitoring for SARS-CoV-2 to non-hospital spaces is an intriguing perspective. However, previous studies reported concentrations of SARS-CoV-2 RNA below the limit of detection for the protocols used. Conte et al. (21) investigated the presence of SARS-CoV-2 RNA in air samples collected in different indoor spaces (e.g., train stations, food markets, shopping centers, etc…) of three Italian cities. Air samples were collected using active sampling on quartz filters. The sampling volume differed from site to site, depending on opening hours of each site. All collected samples tested negative for the presence of SARS-CoV-2.

Limitations of the study include the small number of observations and the high variability between the two sampling sites. As outlined by the Figure 4, this is probably due to differences in the viral load of patients. However, we cannot exclude that other factors may in-fluence the detection performance of the Spore Trap device, such as rooms ventilation or the air dynamics. Finally, we did not test the viability of the collected viral particles, so we cannot assume that these particles could cause an infection. However, the absence of particles in the rooms hosting SARS-CoV-2 negative patients, confirms that these particles are spread by infected subjects only, and their detection in the air is evidence that the environment is hosting at least a SARS-CoV-2 positive subject. Further larger studies are needed to confirm this potential application of the Spore Trap device and to highlight possible pitfalls.

## 5. Conclusion

In conclusion, the Spore Trap technology is effective in detecting airborne SARS-CoV-2 virus with excellent specificity and high sensitivity, when compared to previous reports.

The SARS-CoV-2 pandemic scenario has suggested that indoor air quality control will be a priority in future public health management and will certainly need to include an environmental bio-investigation protocol.

## Data availability statement

The raw data supporting the conclusions of this article will be made available by the authors, without undue reservation.

## Ethics statement

The studies involving human participants were reviewed and approved by the Comitato Universitario di Bioetica, Università degli Studi di Perugia. The patients/participants provided their written informed consent to participate in this study.

## Author contributions

SP, CE, and LeP conceptualized the research project. ET finalized the manuscript. EM, AV, and CL collected patients' data. SM performed statistical analysis. BC, RC, and AM performed laboratory analysis. LuP, FM, SB, ED, and MP supervised data collection. All authors contributed to the article and approved the submitted version.
