# Supplementary material for: Monitoring of indoor bioaerosol for the detection of SARS-CoV-2 in different hospital settings
Source: Front Public Health. 2023 Apr 20;11:1169073. doi: 10.3389/fpubh.2023.1169073 (PMC10157290; doi:10.3389/fpubh.2023.1169073)
Supplement: Supplementary file 1 [file Table_1.DOCX]

|  | **Spore-Trap negative**  **N (%)** | **Spore-Trap positive**  **N (%)** |
| --- | --- | --- |
| SARS-CoV-2 negative | 10 (100) | 0 (0.0) |
| SARS-CoV-2 positive | 11 (30.6) | 25 (69.4) |
|  | | |
| Sensitivity analysis in the SARS-CoV-2 positive group | | |
|  | | |
| Room features |  |  |
| *Double room* | 8 (30.8) | 19 (69.2) |
| *Single room* | 3 (33.3) | 6 (66.6) |
| Respiratory support |  |  |
| *None* | 0 (0.0) | 2 (100) |
| *O_2_-therapy* | 8 (34.8) | 15 (65.2) |
| *Non-invasive ventilation* | 3 (27.3) | 8 (62.7) |
| Wards |  |  |
| *Internal Medicine SGB* | 3 (20.0) | 12 (80.0) |
| *Internal Medicine UH* | 8 (44.4) | 10 (55.6) |
| *Pulmonology* | 0 (0.0) | 2 (100) |

**Supplementary Table 1**

SGB: San Giovanni Battista; UH: University Hospital
